# Angiomyolipoma Have Common Mutations in TSC2 but No Other Common Genetic Events

**DOI:** 10.1371/journal.pone.0024919

**Published:** 2011-09-16

**Authors:** Wei Qin, Vineeta Bajaj, Izabela Malinowska, Xin Lu, Laura MacConaill, Chin-Lee Wu, David J. Kwiatkowski

**Affiliations:** 1 Translational Medicine Division, Department of Medicine, Brigham and Women's Hospital, Boston, Massachusetts, United States of America; 2 Departments of Pathology and Urology, Massachusetts General Hospital, Boston, Massachusetts, United States of America; 3 Center for Cancer Genome Discovery, Dana-Farber Cancer Institute, Boston, Massachusetts, United States of America; Instituto Nacional de Câncer, Brazil

## Abstract

Renal angiomyolipoma are part of the PEComa family of neoplasms, and occur both in association with Tuberous Sclerosis Complex (TSC) and independent of that disorder. Previous studies on the molecular genetic alterations that occur in angiomyolipoma are very limited. We evaluated 9 angiomyolipoma for which frozen tissue was available from a consecutive surgical series. Seven of 8 samples subjected to RT-PCR-cDNA sequencing showed mutations in TSC2; none showed mutations in TSC1 or RHEB. Six of the seven mutations were deletions. We searched for 983 activating and inactivating mutations in 115 genes, and found none in these tumors. Similarly analysis for genomic regions of loss or gain, assessed by Affymetrix SNP6.0 analysis, showed no abnormalities. Loss of heterozygosity in the TSC2 region was commonly seen, except in patients with low frequency TSC2 mutations. We conclude that sporadic renal angiomyolipoma usually have mutations in TSC2, but not TSC1 or RHEB, and have no other common genomic events, among those we searched for. However, chromosomal translocations and gene fusion events were not assessed here. TSC2 inactivation by mutation is a consistent and likely necessary genetic event in the pathogenesis of most angiomyolipoma.

## Introduction

Renal angiomyolipoma (AML) are part of the spectrum of perivascular epithelioid cell neoplasms (PEComas), which are defined as “mesenchymal tumors composed of histologically and immunohistochemically distinctive perivascular epithelioid cells (PEC)” [1], [2]. They have an incidence in the adult population of about 1 in 1,000 normal individuals [3], [4]. Typical AML show triphasic histology, with tortuous, thick-walled blood vessels, irregularly arranged sheets and bundles of myoid-appearing PEC, and lipid-filled PEC that appear to be adipocytes [2]. However, the proportions of these cell types is highly variable, and cases are seen in which there is predominance of any of myoid PEC, lipid-filled PEC, or epithelioid PEC. They are seen in otherwise normal individuals, but occur at much higher frequency in patients with the genetic disease Tuberous Sclerosis Complex (TSC), where the incidence in adults is about 70% [5], [6]. Renal AMLs occurring in TSC are often large, multiple, and bilateral in location, while each of these features is uncommon or rare in non-TSC individuals who have these neoplasms [4]. Indeed, in a recent series from a single institution, none of 32 patients with AML who did not have TSC had bilateral AMLs [7].

Renal AML are often identified as an incidental finding on abdominal imaging studies done for other reasons. Their chief clinical significance is that large lesions are prone to spontaneous hemorrhage, progressive lesions can compromise renal function, and fat-poor AML are difficult to distinguish from other renal neoplasms [4]. The management of renal AMLs can be challenging, and is evolving with the recent recognition that rapamycin may have benefit for this disease [8]. In addition to rapamycin and analogues, current management approaches include selective arterial embolization, radiofrequency ablation, and nephron-sparing surgery [7], [9].

The origin and genetic basis of AMLs is uncertain. AML and other PEComas express several proteins whose expression is typical of melanocytes, including GP100silver, the antigen detected by the HMB-45 monoclonal antibody, suggesting that the developmental or genetic basis of these neoplasms may be related to normal or aberrant melanocyte development [10]. This feature, combined with their smooth muscle cell character, has suggested that they may have a neural crest origin [11]. AMLs occurring in TSC are well-known to show evidence of bi-allelic inactivation of the TSC1 or TSC2 gene, corresponding to the germline mutation present in such individuals [12].

Limited studies have been performed to analyze the genetic basis of AML development in non-TSC individuals. Loss of heterozygosity (LOH) of the TSC2 region has been reported [13], [14], and angiomyolipoma associated with pulmonary lymphangioleiomyomatosis have been shown to contain TSC2 mutations in a small number of cases [15]. However, other angiomyolipoma do not appear to have this finding consistently [16]. More recently, LOH for the TSC2 region was identified in 11 of 12 PEComas, and 6 of 14 ‘classic’ angiomyolipoma [17]. Many reports have documented the consistent activation of the mTOR pathway in these tumors [17], [18], [19], and reduction or absence of TSC2 expression was shown in four cases examined by immunoblotting [19]. Nonetheless, the frequency of TSC1/TSC2 involvement in the development of these tumors has not been clear, due to the small number of cases examined and inconsistency of the findings. In addition, no studies have examined these tumors in any detail for other genetic and genomic changes that may contribute to their development.

Here we describe detailed genetic analyses as well as the clinical features of a consecutive series of 9 angiomyolipoma from a single institution. We find small inactivating mutations in TSC2 in nearly all tumors, and no evidence for other genetic alterations.

## Results

### Samples

**Figure 1:**
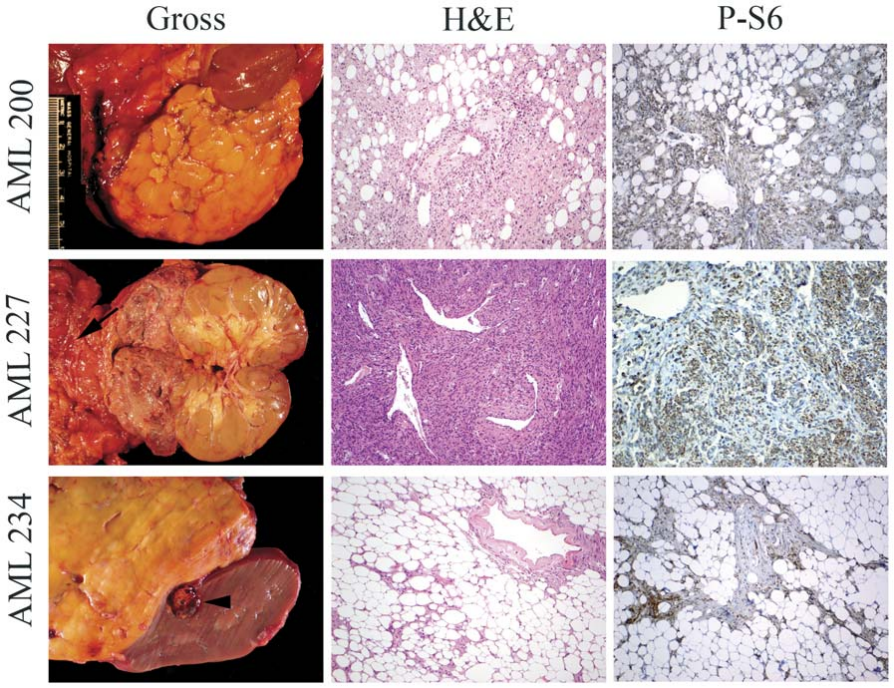
Angiomyolipomas. Gross pictures, micrographs of H&E stains, and immunohistochemical stains with anti-phospho-S6 (S235/S236, P-S6) of AML200, AML227 and AML234 are shown. AML227 arose in the lower pole of the kidney and extended to the attached colon (arrow). AML234 arose adjacent to the kidney. A small renal cell carcinoma is present in the kidney parenchyma (arrowhead).

We studied all AMLs for which frozen tissue was available from resections performed at a large general hospital (Mass. General Hosp.) during a 4 year period. Ages and sex of subjects and other clinical information is shown in Table 1. Nine specimens from 9 patients were available, of whom 1 had a diagnosis of Tuberous Sclerosis Complex (TSC), and 1 had a diagnosis of Von Hippel Lindau (VHL) disease. The remaining 7 patients had no clinical features of TSC other than renal AML, or other known genetic disease. The tumors all had classic diagnostic features of AML, though there was a highly variable fat/adipocyte and spindle cell components, and two had predominant epithelioid morphology (Table 1, Figure 1). Immunohistochemistry, including staining for HMB45, was performed when indicated to confirm the diagnosis (not shown). All AMLs expressed high levels of phospho-S6-S235/S236 (P-S6), indicative of activation of mTORC1, as we and others have reported previously [17], [18], [19] (Figure 1). This finding is consistent with inactivation of TSC1/TSC2 in these tumors.

**Table 1:** Clinical features of patients with AMLs.

Sample	Age	Sex	Size*	Location	Histology	Year	Genetic diagnosis
AML200	69	F	8.5 cm	Surface of R kidney	Triphasic	2005	
AML227	37	F	8 cm	R kidney, extending into retroperitoneum and mesenteric fat	Spindle cell rich	2006	
AML234	47	F	19 cm	L extra-renal mass	Fat-rich, concurrent renal cell carcinoma	2006	
AML291	44	M	4.5 cm	R Kidney	Epithelioid	2007	
AML400	58	M	3.1 cm	R Kidney	Triphasic	2008	VHL
AML403	45	M	1.7 cm	L Kidney	Triphasic	2008	
AML406	40	F	15 cm	L kidney, extending into retroperitoneum	Triphasic	2008	TSC
AML465	47	F	4.9 cm	R Kidney	Epithelioid	2008	
AML490	53	F	2.5 cm	L Kidney	Triphasic	2009	

*largest dimension.

Triphasic denotes classic AML histology, with myoid, adipocyte, and vascular elements.

R, right; L, left.

### Mutational analysis of *TSC1*, *TSC2*, and *RHEB*

**Figure 2:**
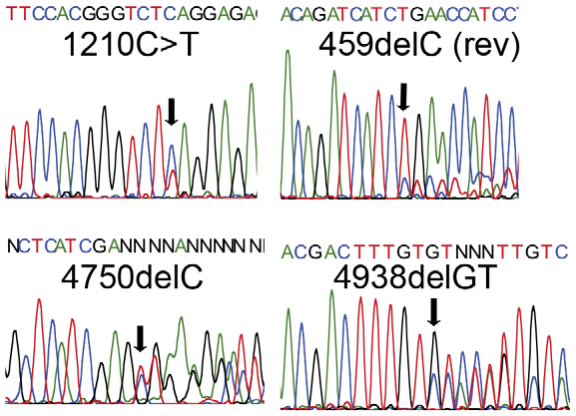
Representative sequencing traces demonstrating TSC2 mutations. A–D. Mutations in TSC2.

Using RNA preparations and RT-PCR, cDNA sequencing was performed for the entire coding region of *TSC1*, *TSC2*, and *RHEB*. Seven of eight AML samples sequenced had a total of 5 distinct deletion and 1 nonsense mutation in *TSC2* (Table 2, Figure 2A–D). All seven were confirmed by analysis of genomic DNA from the same frozen tumor material, and 6 of 7 were confirmed in paraffin block extracted DNA preparations from these same tumors (Table 2). The mutation found in one sample (AML234) could not be detected in genomic DNA from samples from paraffin blocks of the same tumor, possibly due to the large size of that AML (19 cm diameter) and the possibility that it had occurred by fusion of separate independent AMLs with distinct molecular lesions. One of the seven AML TSC2 mutations (AML406) was also seen in normal tissue DNA from the same patient. This was the patient who had TSC, consistent with it being the germline TSC2 mutation in that individual. The other six AML mutations identified in TSC2 were not seen in normal tissue samples from the corresponding patients, consistent with their occurrence as somatic events in the AMLs.

**Table 2:** Summary of genetic findings in AML patients.

Sample	TSC1, TSC2MLPA	TSC2LOH	Sequencing results			Onco- map	SNP6.0AffyArray
			TSC1	TSC2	RHEB		
AML200	Neg	NO	Neg	c.5238–5255delp.(His1746_Arg1751delinsGln)	Neg	Neg	Negative
AML227	Neg	YES	Neg	c.1210C>Tp.(Gln404X)	Neg	Neg	ND
AML234	Neg	YES	Neg	c.459delC*	Neg	Neg	ZNF761 amplification
AML291	TSC1-E1 amplif.†	YES	ND	ND	ND	Neg	ND
AML400 (VHL)	Neg	NO	Neg	Neg	Neg	ND	Negative
AML403	Neg	YES	Neg	c.4750delC*	Neg	ND	Negative
AML406 (TSC)	Neg	YES	Neg	c.5238–5255delp.(His1746_Arg1751delinsGln)	Neg	Neg	SUPT3H deletion
AML465	ND	NO	Neg	c.682–715del34*	Neg	ND	Negative
AML490	ND	NO	Neg	c.4938–4939delGT*	Neg	ND	C7orf10 deletion

*Not previously reported (per LOVD Tuberous sclerosis database for TSC2, chromium.liacs.nl/LOVD2/TSC).

† Amplif. denotes amplification, with three copies detected for exon 1.

ND, not done. Not all studies were performed on all samples due to limitations in amounts of material.

TSC2 mutations were not identified by cDNA sequencing in one AML patient. This patient had a diagnosis of von Hippel Lindau (VHL) Syndrome, with a germline c.250G>T (p.Val84Leu) VHL mutation. Analysis of the AML sample from that VHL patient indicated that there was equivocal evidence of loss of the wild type allele of VHL (data not shown). Similarly, there was no clear evidence of loss of heterozygosity (LOH) findings for markers within TSC2 (data not shown). This may reflect absence of LOH in this sample, or alternatively low AML purity.

No mutations were detected in *TSC1* or *RHEB*. RT-PCR of *RHEBL1* failed on all samples, suggesting that its level of expression was very low to nil in AMLs.

Multiplex ligation-dependent probe assay (MLPA) and LOH analysis using 3 microsatellite markers was used to assess changes in copy number in the *TSC1* (MLPA only) and *TSC2* genes in these samples. Copy number was normal for all exons of TSC2 in all samples, while one sample showed evidence for duplication of *TSC1* exon 1. This observation was verified by repeat analysis using a different set of MLPA probes (data not shown), but the significance of this finding is uncertain since exon 1 is a non-coding exon. LOH analyses demonstrated that 5 of 9 samples had evidence for reduction in allele signal (LOH) for one or more informative microsatellite markers near TSC2 (data not shown). In the other four cases, LOH was not seen. However, the representation of the mutant TSC2 allele was relatively low in those four cases, suggesting significant admixture with normal cells in those AML specimens, making it difficult or impossible to discern LOH using microsatellite markers.

### Absence of other common cancer mutations or genomic deletions/amplifications in AMLs

We searched for other mutations (983 total) in a set of 115 common cancer genes by mass spectrometry [20] on five of these AML samples. No mutations were identified in any gene studied by this method.

Seven AMLs, for which sufficient quality and quantity of DNA were available, were analyzed using Affymetrix SNP6.0 arrays to assess copy number of genomic fragments across the genome, and search for regions of deletion or amplification. Multiple regions of potential copy number variation were seen, but the nearly all aligned with regions known to be variable in copy number in the human population. Three possible sites of regional, specific deletion or amplification were seen: amplification of *ZNF761* (chr 19, 90 kb) in AML234; deletion of *SUPT3H* (chr 6, 160 kb) in AML406; and deletion of *C7orf10* (chr 7, 160 kb) in AML490. All three of these sites were examined by MLPA using duplicate probe sets for each genomic region, in both AML and normal DNA samples. The sites of amplification and deletion were confirmed, respectively, in the AML DNA samples (data not shown). However, they were also seen to be present in normal kidney tissue from each patient, suggesting that they were sites of germline copy number variation, and were not somatic events contributing to AML development.

## Discussion

Similar to past series, there was a small female preponderance in the cases of angiomyolipoma analyzed here, with 6 of 9 being female [3]. In addition, seven of the nine cases had no clinical evidence of genetic disease, reflecting the common clinical occurrence of this tumor in sporadic form. Further, for 6 of the 7 cases in which a TSC2 mutation was found in the AML, this mutation was not present in normal tissue DNA, indicating that they were not germline carriers of a TSC2 mutation. The one patient in which the TSC2 mutation was present in normal tissue had TSC, as expected.

We found small mutations in TSC2 in 7 of 8 angiomyolipoma that were sequenced, and the single case that did not harbor a mutation was the VHL patient. No mutations were identified in either TSC1 or RHEB, consistent with previous reports [16]. Interestingly, 6 of 7 mutations in TSC2 were deletions, which is significantly different from the collective TSC2 mutation database experience on germline TSC2 mutations [21], for which deletions account for only 27% of all TSC2 mutations (P = 0.013, Chi-square test). This may reflect differences in mutational mechanisms that occur in somatic vs. germline tissues. Alternatively it may indicate that deletion mutations have a stronger effect than missense or other types of mutations in complete elimination of TSC2 GAP activity toward RHEB. Concordant with these mutation findings in TSC2, TSC2 LOH was also seen in 5 AMLs; tumor impurity may have contributed to the lack of TSC2 LOH in the other 4 cases. In every case in which TSC2 LOH was seen, neither MLPA nor the SNP6.0 array indicated copy number reduction in the region of TSC2, suggesting that copy neutral LOH had occurred, with loss of one copy of chromosome 16p and replacement with the other copy including a TSC2 mutation. Overall, these data indicate that TSC2 inactivation is a consistent and likely necessary genetic event in the pathogenesis of most angiomyolipoma.

These observations on the consistent involvement of TSC2 in the pathogenesis of AML fit with previous observations on the high frequency of activation of the mTORC1 pathway in this disease [16], [18], [19]. The TSC1/TSC2 protein complex is known to have a critical role in the regulation of the state of activation of mTORC1, by acting as a GAP (GTPase activating protein) for Rheb [24], [25]. Complete loss of either TSC1 or TSC2 from cells causes strong activation of mTORC1 with downstream phosphorylation effects that are critical for the ensuing growth of these tumors.

The occurrence of AML in VHL has not been reported previously. This co-occurrence may be due to chance alone. However, the lack of a TSC2 point mutation in this single case suggests the possibility that there is a different molecular pathogenesis in this circumstance.

Our broader survey for common mutational events gave no positive findings in AML. None of 983 common cancer point mutations were identified. In addition, there was no evidence for genomic deletions or amplifications, that would have been detected as copy number changes in the SNP analysis. Balanced translocations and interstitial rearrangements would not be detected by these methods, and may be present in these tumors. More detailed genomic studies to investigate this possibility, such as whole genome sequencing, are appropriate.

## Materials and Methods

### Angiomyolipoma

Angiomyolipoma tissue samples were obtained at the time of surgical resection from patients who were operated on at the Massachusetts General Hospital (MGH) during the period 2005–2009. This study was approved by the Partners Human Research Committee, the Institutional Review Board for our hospitals. All patients were included from whom tumor samples were large enough to permit collection of frozen tumor. Frozen tissue from adjacent normal kidney was also available from three patients. Frozen tissues were stored at −80°C until use. AML samples were used for both DNA and RNA extraction, by standard methods.

Paraffin cores of AMLs and normal kidney were also used for extraction of DNA. Paraffin punch cores were chopped into small pieces with a feather scalpel in tubes, and DNA was extracted using the QIAamp DNA FFPE Tissue Kit (Qiagen) according to the manufacturer's instructions.

### RNA analyses

Reverse transcription and PCR (RT–PCR) were performed using QIAGEN OneStep RT-PCR kit (QIAGEN, Germany) on RNA isolated from the AML tissues and available normal tissues. The *TSC1* and *TSC2* cDNAs were amplified using 6 and 8 pairs of cDNA primers, respectively, yielding products of size 296–797 nt (Table S1). Similarly, *RHEB* and *RHEBL1* cDNAs were amplified using a single primer pair to yield products of size 630 and 656 nt, respectively. Following PCR amplification of cDNA, fragments were separated by agarose gel electrophoresis, purified using the QIAquick Gel Extraction kit (QIAGEN, Germany), and then sequenced bidirectionally by standard Sanger sequencing with fluorescent dideoxynucleotides. Mutations identified in this manner were confirmed by Sanger sequencing of DNA extracted from the frozen tumors as well as from paraffin-embedded specimens, including normal control tissue.

### Multiplex ligation-dependent probe assays (MLPA) and loss of heterozygosity (LOH) analysis with microsatellite markers

AML DNA samples were examined for genomic deletions in *TSC1* and *TSC2* using MLPA including probe sets for each of the exons of *TSC1* and *TSC2*, as described previously [22]. Three microsatellite markers in the region of the TSC2 gene were employed for LOH analyses, and details of the markers, heterozygosity, genomic location, and primer sequences are given in Table S2. Genotyping of these markers was performed using fluorescently tagged oligonucleotides in PCR, followed by capillary electrophoresis on the ABI 3730. Allele ratios were quantified using GeneMapper version 3.0 (Applied Biosystems).

### Oncomap analysis

We performed mass spectrometry analysis on these samples (Oncomap), performing 1047 assays interrogating 983 unique mutations in 115 genes [20]. All positive findings from an initial multiplex screen were followed up with a more sensitive and robust singleplex (HME) analysis on the Sequenom MassARRAY platform (San Diego, CA).

### Microarray analysis

AML samples were analyzed for copy number variation using Affymetrix SNP 6.0 arrays in the Microarray Core facility at Dana Farber Cancer Institute. Results were compared to that of four normal DNA samples from unrelated individuals. DNA copy number data was segmented into regions of estimated equal copy number using circular binary segmentation (CBS) as implemented in GenePattern. DNA copy number data as well as segmented data was visualized in IGV [23] to identify chromosomal regions of copy number loss and gain, and were compared with known copy number variable regions identified in previous studies of non-tumor DNA.

### Immunohistochemistry

Tissue sections (5 µm) were prepared from formalin-fixed, paraffin-embedded renal AML tissue blocks, corresponding to the frozen AMLs, for immunohistochemical analysis using the avidin-biotin complex (ABC) method. Briefly, slides were deparaffinized and sections were heated in a pressure cooker in 10 mM citrate buffer (pH 6.0) for antigen retrieval. Endogenous peroxidase activity was blocked by incubation in 3% H2O2 for 20 min at room temperature. 5% normal goat in Tris-buffered saline-Tween (TBST) with Avidin was used for blocking. Antibodies to phospho-S6 (Ser235/236;Cell Signaling Technology #2211; 1∶200 dilution) or Clone HMB-45 (Dako IR052, 1∶50 dilution) diluted in 5% normal goat in TBST with Biotin were applied and incubated overnight at 4°C. Biotinylated goat anti-rabbit secondary antibody (Vector Labs; 1∶300 dilution) was applied for 30 min prior to ABC-complex. Negative controls included adjacent sections incubated in TBST without primary antibody. Immunodetection was performed with Liquid DAB (DAKO). Hematoxylin was used as a counterstain, and an adjacent section was stained with hematoxylin and eosin.

## Supporting Information

Table S1 RT-PCR primer sequences.
(DOC)

Table S2 Microsatellite markers for TSC2.
(DOC)
