# Supplementary material for: Angiomyolipoma Have Common Mutations in TSC2 but No Other Common Genetic Events
Source: PLoS One. 2011 Sep 16;6(9):e24919. doi: 10.1371/journal.pone.0024919 (PMC3174984; doi:10.1371/journal.pone.0024919)
Supplement: Table S1 — RT-PCR primer sequences. (DOC) [file pone.0024919.s001.doc]

Supplemental Table S1. RT-PCR primer sequences

| **Amplicon** | **cDNA primer** | **Product length** |
| --- | --- | --- |
| TSC1-1 | AACCTGTAGCACACGTCCTG | 722 |
|  | CCATCATTGGCTTGACCACT |  |
| TSC1-2 | GAAAGAAAACCTGGAGACTTTTGA | 719 |
|  | CAGGTGGCTCTTCTGATCCT |  |
| TSC1-3 | CAACACCATCTTCTGAATGACAG | 751 |
|  | AAGGAGGAGAGCCTCCAAAG |  |
| TSC1-4 | GCCTTTACCCAGCAAGTCTG | 724 |
|  | CCGTTTTTGGGAGGTATCAA |  |
| TSC1-5 | AGCCATGTTCTCCAGCAGAC | 700 |
|  | CCGTTTTTGGGAGGTATCAA |  |
| TSC1-6 | GGCATGACCAGTAGCCTTTC | 296 |
|  | GTCCCATTTCCACACATGAA |  |
| TSC2-1 | GCCAAACCAACAAGCAAAGA | 690 |
|  | CAGGCAGTTGTAGCAGACCA |  |
| TSC2-2 | GGTCTCCCTGCAGGTGCT | 725 |
|  | GGGACTCGCTCCTGAAGAAT |  |
| TSC2-3 | ACGGCTGGATTCAGAACCT | 757 |
|  | CGGAAGAGCAGGGAGTAGG |  |
| TSC2-4 | CCCTTTCTCCTCCCACAGG | 713 |
|  | CCTGATGAACCACATGGCTA |  |
| TSC2-5 | CACCAACCCCTCCAAGTTTA | 725 |
|  | CAGCCTGGGACTCCAGCTT |  |
| TSC2-6 | GCATGTGAGACAGACCAAGG | 713 |
|  | AGGACGACCTGCTGTAGGC |  |
| TSC2-7 | AGCGCTAGGCATGGACAG | 749 |
|  | TGTACCTGTAGGAGCCATGC |  |
| TSC2-8 | CTCGCCATCCTGTCCAAT | 797 |
|  | GACAGGCAATACCGTCCAAG |  |
| RHEB | AAGATGCCGCAGTCCAAGT | 630 |
|  | CTTCAAGGAGAACGGGCAGT |  |
| RHEBL1 | CTCACACCCTGACCTCGTTT | 656 |
|  | GCAGAAGCAAGGCAGTTACC |  |
