# Supplementary material for: Angiomyolipoma Have Common Mutations in TSC2 but No Other Common Genetic Events
Source: PLoS One. 2011 Sep 16;6(9):e24919. doi: 10.1371/journal.pone.0024919 (PMC3174984; doi:10.1371/journal.pone.0024919)
Supplement: Table S2 — Microsatellite markers for TSC2. (DOC) [file pone.0024919.s002.doc]

Supplemental Table S2. Microsatellite markers for TSC2.

| Name | Genomic position on chr 16  (hg19) | Size range | Repeat  Unit | Hetero-zygosity | Oligonucleotide primer sequences |
| --- | --- | --- | --- | --- | --- |
| STR3 | 1880918 | 124-142 | GT | 70% | | CCTTCCTCCCCGCCATATATGTGTA | | --- | | GCAGTCACCATCCCGACGCA | |
| kg8 | 2138867 | 117-129 | GT | 60% | | GCCAGCTCCGAGGGCCTTGA | | --- | | GACTCCTCCTGGGGGCTGGC | |
| STR7 | 2393710 | 116-130 | CTAT | 70% | | GCTCCCAATTCTTTGAAGGGGTACAA | | --- | | TCAGCAATTTGGGTACTGTTGTACGAT | |
